# Airflow and Particle Deposition in Acinar Models with Interalveolar Septal Walls and Different Alveolar Numbers

**DOI:** 10.1155/2018/3649391

**Published:** 2018-09-25

**Authors:** Jinxiang Xi, Mohamed Talaat, Hesham Tanbour, Khaled Talaat

**Affiliations:** ^1^Department of Biomedical Engineering, California Baptist University, Riverside, CA 92504, USA; ^2^Department of Aerospace, Industrial, and Mechanical Engineering, California Baptist University, Riverside, CA 92504, USA; ^3^School of Engineering and Technology, Central Michigan University, Mt. Pleasant, MI 48858, USA; ^4^Department of Nuclear Engineering, The University of New Mexico, Albuquerque, NM 87131, USA

## Abstract

Unique features exist in acinar units such as multiple alveoli, interalveolar septal walls, and pores of Kohn. However, the effects of such features on airflow and particle deposition remain not well quantified due to their structural complexity. This study aims to numerically investigate particle dynamics in acinar models with interalveolar septal walls and pores of Kohn. A simplified 4-alveoli model with well-defined geometries and a physiologically realistic 45-alveoli model was developed. A well-validated Lagrangian tracking model was used to simulate particle trajectories in the acinar models with rhythmically expanding and contracting wall motions. Both spatial and temporal dosimetries in the acinar models were analyzed. Results show that collateral ventilation exists among alveoli due to pressure imbalance. The size of interalveolar septal aperture significantly alters the spatial deposition pattern, while it has an insignificant effect on the total deposition rate. Surprisingly, the deposition rate in the 45-alveoli model is lower than that in the 4-alveoli model, indicating a stronger particle dispersion in more complex models. The gravity orientation angle has a decreasing effect on acinar deposition rates with an increasing number of alveoli retained in the model; such an effect is nearly negligible in the 45-alveoli model. Breath-holding increased particle deposition in the acinar region, which was most significant in the alveoli proximal to the duct. Increasing inhalation depth only slightly increases the fraction of deposited particles over particles entering the alveolar model but has a large influence on dispensing particles to the peripheral alveoli. Results of this study indicate that an empirical correlation for acinar deposition can be developed based on alveolar models with reduced complexity; however, what level of geometry complexity would be sufficient is yet to be determined.

## 1. Introduction

Alveolar sacs are the ends of the respiratory tree and the smallest respiration units. The alveoli have a complex framework and are supported by interalveolar septa [1]. Pores of Kohn are apertures in the alveolar septum, which are circular or oval in shape and allow communications among adjacent alveoli [2]. This collateral ventilation helps equalize pressures across alveoli and plays an important role in preventing lung collapse (i.e., atelectasis) [3] and promoting alveolar recruitment [4]. In the case of emphysema, the number and size of pores increase in early stages [5–7]. In later stages, destruction of septa and even framework breakdown have been observed, leading to a decrease of the elasticity and an increase of the sac airspace [8]. Aging can also cause the breakdown of interalveolar septa and increase the number and sizes of alveolar pores of Kohn, thereby decreasing the collateral flow resistance among alveoli [9, 10]. Understanding the effects of alveolar septum and pore on alveolar airflow and particle dynamics is fundamental for understanding the pathology of pulmonary diseases, improving ventilator management, and devising more effective treatment strategies.

Due to the extremely large number of alveoli in the lungs (∼480 million), it is impossible to construct a complete model of the acinar airspace. Even to construct a single acinar unit will be a formidable task, considering that each acinar unit consists of more than 10,000 alveoli [1]. As a result, alveolar models with varying realism and complexities had been proposed to understand particle dynamics inside the alveoli. These included alveolus models comprising a single hemisphere attached to a duct (i.e., respiratory bronchiole) [11–16], an alveolar duct with multiple alveoli (i.e., alveolated duct) [17, 18], and space-filling-based models with honeycomb or polygonal structures (i.e., terminal alveolar sacs) [19–23]. Based on *in vivo* microscopy observations, Kitaoka et al. [19] proposed that the alveolar mouth closes at minimum volume and gradually opens during inhalation. Based on a series of acinar models, Khajeh-Hosseini-Dalasm and Longest [22] investigated the effect of geometry complexity on acinar deposition. It was observed that when the acinar models consisted of more than three alveolar duct generations, the total acinar deposition rates were similar among models and were not affected by gravity orientation either. However, the effects of pores of Kohn on acinar deposition remain unclear and underinvestigated. More recently, Hofemeier et al. [23] proposed an algorithm to construct generic heterogeneous acinar models ranging from 372 to 2361 alveoli. They observed that variance in acinar heterogeneity played a minor role in determining alveolar deposition while the deposition rate increased for deeper inhalations.

General findings from previous studies are summarized as follows: classical alveolar deposition correlations are less accurate and need to be improved; wall motion is essential in determining the alveolar flows and aerosol dynamics; geometry complexity and realism strongly affect the predictions of alveolar deposition. Classical alveolar deposition correlations were typically based on particle sedimentation in steady tubular flows [24, 25] and used analytical approximations of particle deposition mechanisms [26]. Such corrections neglected a number of factors that are crucial in accurately determining particle transport and deposition, such as tidal breathing, wall motion, and geometry details. Kojic and Tsuda [27] showed that using steady-flow solutions to approximate oscillatory flows underestimated local particle deposition densities, and this error increased quickly for increasing oscillation frequencies. When the oscillation period became equivalent to the characteristic time for gravitational sedimentation, particle deposition would no longer be approximated by the classical solution based on steady flows as proposed in [28]. Alveolar wall motion has been demonstrated to be essential to match single-path-transport model predictions with *in vivo* alveolar deposition data [29, 30]. It is noted that wall kinematics, such as symmetric vs. anisotropic oscillations, played a negligible role in the alveolar deposition [15, 31]. On the contrary, the type and complexity of the acinar airway yield unique features of airflow and particle transport patterns. Kumar et al. [20] simulated airflow in acinar models with honeycomb structures and reported recirculation inside the alveoli induced by oscillatory wall motions. Talaat and Xi [15] numerically investigated aerosol deposition in a single terminal alveolus with rhythmical oscillations and found significantly different particle dynamics in comparison to that in alveolated ducts or respiratory bronchioles [12, 21, 32, 33]. Particles move back and forth driven by the oscillating walls of the terminal alveolus and form multifolding trajectories [15]; by contrast, particles in an alveolated duct or respiratory bronchiole geometry remain suspended in the alveolus for several breathing cycles, rotating clockwise during exhalation and counterclockwise during inhalation [12, 21].

Several controversial observations have been reported regarding the influences from gravity orientation angle, airway realism, and breathing depth. Haber et al. [12] and Sznitman et al. [21] reported that particle deposition efficiencies are strongly related to the gravity orientation in both alveolated ducts and space-filling geometries. By contrast, Khajeh-Hosseini-Dalasm and Longest [22] suggested that total acinar deposition rates were insensitive to the gravity orientation when the geometry had more than three alveolar duct generations. While some studies [18, 34, 35] found that the geometry complexity significantly affected acinar aerosol deposition, Hofemeier et al. [23] recently observed that heterogeneity in acinar geometry had little effect on alveolar deposition. Similarly, while Hofemeier et al. [23] reported that the acinar deposition rate increased for deeper inhalations, Talaat and Xi [15] reported that the deposition was relatively insensitive to the breathing depth in single terminal alveolar models.

Unique features exist in the acinar airspace. Structurally, there are septal walls and pores of Kohn between neighboring alveoli, which are expected to strongly affect airflow and particle deposition, but whose influences have rarely been studied. Dynamically, the contribution of particle-wall interception to deposition can be important due to the geometrical complexity. But its importance relative to other deposition mechanisms, such as oscillatory convection, gravitational sedimentation, and particle dispersion, is unclear. Moreover, interalveolar septa and pores of Kohn can change in shape and size due to diseases or aging. As a result of these complexities, most space-filling-based honeycomb or polyhedral models to date have neglected the interalveolar septa and pores.

The objective of this study is to investigate the effects of acinar geometrical details, such as the interalveolar septum and pore size, on acinus airflow and particle dynamics in both a simplified 4-alveoli model and a 45-alveoli model. There are four specific aims in this study: (1) to develop acinar models with different number of alveoli and with septa and pores of different sizes, (2) to characterize airflows and particle motions in these acinar geometries, (3) to quantify the effect of pore size on surface doses both temporally and spatially, and (4) to evaluate the acinar deposition in simplified and complex models under the influence of the following factors: particle size, geometrical complexity, gravity orientation angle, and inhalation depth.

## 2. Methods

### 2.1. Study Design

Pulmonary alveoli are composed of a myriad of alveolar sacs arranged in a fractal manner. To study particle deposition in these regions, we started with a simplified four-sac alveolar model, with septal walls and pores of Kohn between adjacent sacs. Due to its well-defined shape and dimension, this model allowed controlled testing of influential parameters such as particle diameter, breathing conditions, and pore sizes. As a result, a comprehensive understanding of deposition mechanisms in alveolar sacs can be obtained. By doing so, airflow and particle dynamics in a control case with rhythmic wall motions were examined; the resulting particle deposition was characterized both temporally (dynamic deposition growth) and spatially (in each alveolus). Six controlled tests were then conducted. To study the effects of rhythmic wall motions, Test 1 compared particle deposition in alveolar models with dynamic and rigid walls. For the model with rigid walls, the flow was stagnant (i.e., zero velocity), and particles moved due to gravity. Test 2 investigated the effect of particle size on alveolar deposition, which was 0.5 *µ*m, 1 *µ*m, 2 *µ*m, and 3 *µ*m. Test 3 investigated alveolar deposition under four gravity orientation angles (0°, 45°, 90°, and 135° from the gravity). The effects of breath-holding and respiration depth were studied in Tests 4 and 5, respectively. The breath holding durations included 0.5, 1, 2, 3, and 4 seconds following the inhalation. The respiration depths included 1, 2, 3, and 4 times of the standard tidal volumes, which is 23.3% of the functional residual capacity (FRC). The last test (Test 6) studied the effects of pore sizes on particle deposition that included three different pore sizes and one model without the septal wall.

A more complex and physiologically realistic model was then developed that contained 45 alveolar sacs. Airflow and particle deposition in this model were compared to the simplified 4-alveoli (or 4-sac) model to determine the feasibility of using simplified alveolar models for inhalation dosimetry predictions and to evaluate the impact from gravitational orientation and inhalation depth. Model development, fluid-particle tracking algorithms, and numerical methods are detailed below.

### 2.2. Computational Acinar Model and Kinematics

**Figure 1:**
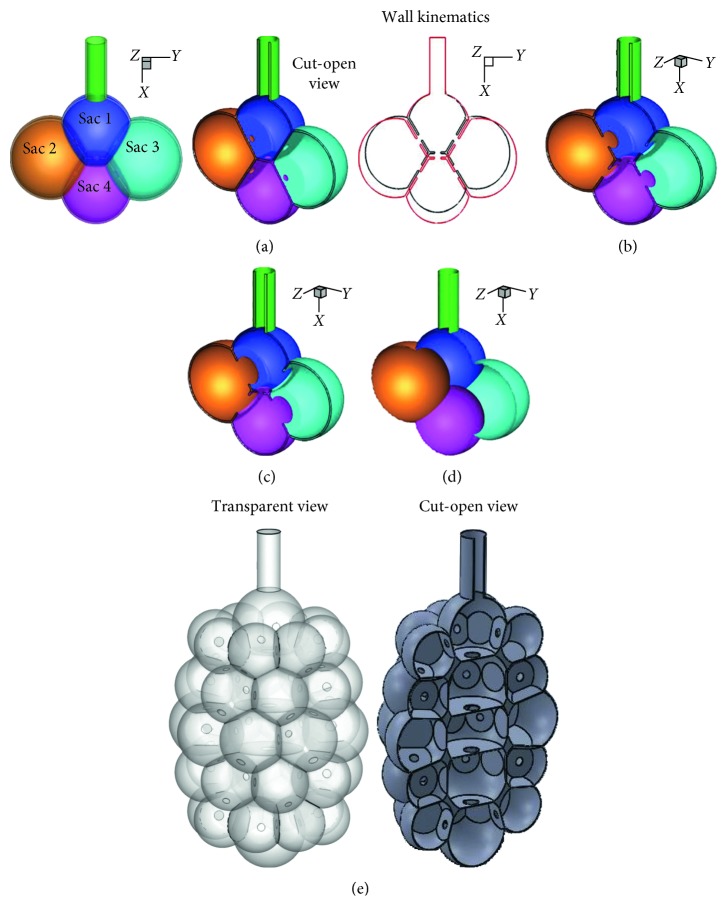
Simplified and complex multiple-alveoli models with septa and pores: (a) simplified 4-alveoli (or 4-sac) model with pores (septal apertures) of 40 *μ*m in diameter; (b) 4-alveoli model with 100 *μ*m pores; (c) 4-alveoli model with 160 *μ*m pores; (d) 4-alveoli model with no spectrum; and (e) 45-alveoli model with 40 *μ*m pores.

To model an acinar cluster, four alveoli were retained in the simplified model. Individual alveolus was approximated using a 0.3 mm diameter sphere. The alveolar cluster was joined to a duct with a length of 0.2 mm and a diameter of 0.1 mm. The left panel of Figure 1(a) shows the air-filled geometry of the above duct-alveoli model, while the middle panel of Figure 1(a) shows the cut-open view of the hollow duct-alveoli model. There were openings (i.e., pores of Kohn) connecting any two neighboring alveoli, and thus totally five pores existed in this model geometry (Figure 1(a), middle panel). To facilitate later reference to the four alveolar sacs, the upper alveolus was termed as Sac 1, the lower alveolus as Sac 4, and the left and right alveoli as Sac 2 and Sac 3, respectively (Figure 1(a), left panel).

The wall kinematics of the acinar model followed the anisotropic motion of the chest [36, 37], which reported a smaller expansion in the arm-arm (*z*) direction than the head-foot (*x*) and back-front (*y*) directions (i.e., *z* : *y* : *x* = 0.375 : 1 : 1). Under normal breathing conditions, the volume expansion was assumed to be the standard tidal volume, that is, *V*_T_/FRC = 0.233, where *V*_T_ represents the standard tidal volume [38]. A user-defined function (UDF) was written that specified the oscillatory wall motions (right panel of Figure 1(a)). More details of the UDF can be found in Talaat and Xi [15].

To investigate the effects of pore sizes on alveolar deposition, three geometrically similar models with different pore sizes were developed, that is, 40 *µ*m, 100 *µ*m, and 160 *µ*m, as shown in Figures 1(a), 1(b), and 1(c), respectively. For comparison purposes, an alveolar model with no septal wall was also developed (Figure 1(d)).

To investigate the model complexity effects, the second model consisted of 45 alveoli. Similar to the 4-alveoli model, septal walls existed between contiguous alveoli and pores of Kohn existed in the septal walls (Figure 1(e)). Even though spheres were initially used to approximate the alveoli, they naturally evolved into polygonal shapes when multiple spheres intersected each other (Figure 1(e)). The airway volume is 4.32 × 10^−11^ m^3^ for the 4-alveoli model and 4.20 × 10^−10^ m^3^ for the 45-alveoli model in comparison to 1.48 × 10^−11^ m^3^ for the single alveolus model in Talaat and Xi [15].

### 2.3. Airflow and Particle Transport Models

Particles ranging from 0.5 to 3 *µ*m in diameter were investigated because smaller submicron particles deposited in the pulmonary region with different mechanisms (i.e., diffusion), and larger micrometer particles were captured by the upper respiratory tract and could not reach the pulmonary acinus. For each numerical test, multiple (3–24) respiration cycles were modeled, with the first cycle to create the unsteady flow field. An amount of 10,000 particles was inhaled at 0.20 s of the second cycle to simulate the inhalation of a bolus of pharmaceutical particles and was tracked until all particles deposited or exited the geometry with the expiratory flow.

The airflow was isothermal and incompressible in this study. The flow regime is laminar because the Reynolds number is much smaller than one even during peak inhalations [39]. Therefore, the laminar flow model was used to solve the airflow field. A well-tested direct Lagrangian algorithm was used to track particle motions [40, 41]. This algorithm, enhanced by the near-wall treatment algorithm [42], has been shown in our previous studies to agree with *in vitro* deposition results in human upper airways for both nanoparticles [43] and micrometer particles [44, 45].

### 2.4. Numerical Methods

ANSYS Fluent (Canonsburg, PA) with dynamic mesh and discrete phase models was used to simulate the transient airflow and particle deposition. User-defined Fortran and C modules were used to specify alveolar wall kinematics and calculate temporal and spatial surface deposition rates [46, 47]. ANSYS ICEM CFD (Ansys, Inc) was utilized for computational mesh generation. One-way coupling from the airflow to particles was assumed. A grid sensitivity analysis was conducted by testing a range of mesh densities, and grid independent result was considered to be achieved when the difference in total particle deposition was less than 1%. The final mesh was chosen to be 1.2 million cells for the 4-alveoli model and 6.0 million for the 45-alveoli model.

## 3. Results

### 3.1. Airflow Field and Particle Motion

**Figure 2:**
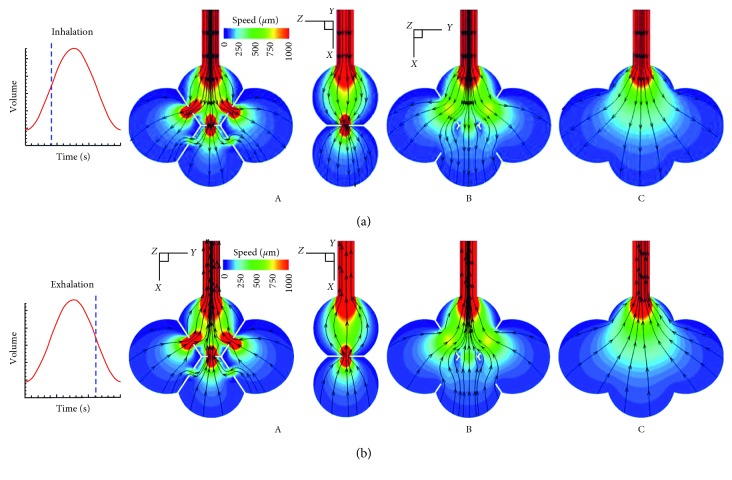
Airflow contour and stream traces in the 4-alveoli models with a pore size of (A) 40 *µ*m, (B) 160 *µ*m, and (C) no septal wall during (a) inhalation and (b) exhalation.

Instantaneous airflow fields at the middle of inhalation and exhalation cycles are shown in Figure 2 in the 4-alveoli model with different pore sizes. Under the normal tidal breathing condition (23.3% FRC, or 1 *V*_T_), the peak velocity at the inlet is around 1 mm/s. In the model with a pore size of 40 *µ*m ((A) in Figure 2), Venturi effect at the pores was observed, which increased the penetration depth of particles into the peripheral alveoli. This effect, however, was absent when the pore sizes were large ((B) in Figure 2) or when the septal wall was missing ((C) in Figure 2). Overall symmetric flow patterns were noted for all of the three models considered (at their peak inhalation and exhalation speeds herein). Interesting discrepancies were also discerned among the three models. In (A) in Figure 2, streamlines flowed from the lower alveolus to the two lateral alveoli during inhalation and reversed their directions during exhalation. By contrast, streamlines in (B) in Figure 2 flowed from the two lateral alveoli to the lower one during inhalation and vice versa. These collateral ventilations were presumably associated with the pressure imbalance between neighboring alveoli.

**Figure 3:**
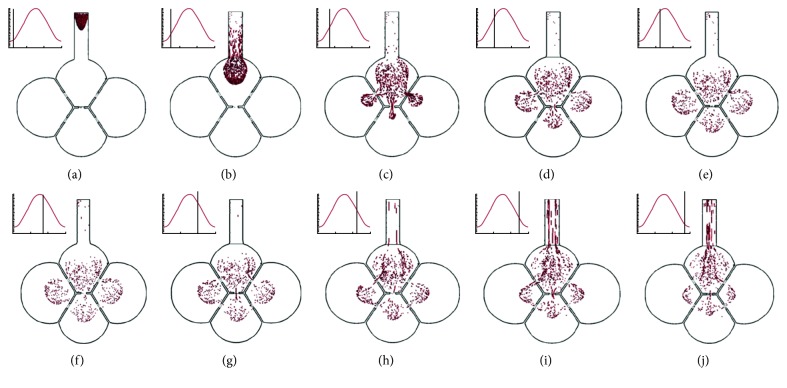
Instantaneous snapshots of particle positions in the 4-alveoli model during the first cycle after particle inhalation. Particles were 1 *µ*m in diameter. Due to particle dispersion, some particles exited the geometry with expiratory airflow. (a) *T* = 0.25 s. (b) *T* = 0.50 s. (c) *T* = 0.75 s. (d) *T* = 1.00 s. (e) *T* = 1.25 s. (f) *T* = 1.75 s. (g) *T* = 2.00 s. (h) *T* = 2.25 s. (i) *T* = 2.50 s. (j) *T* = 2.75 s.

**Figure 4:**
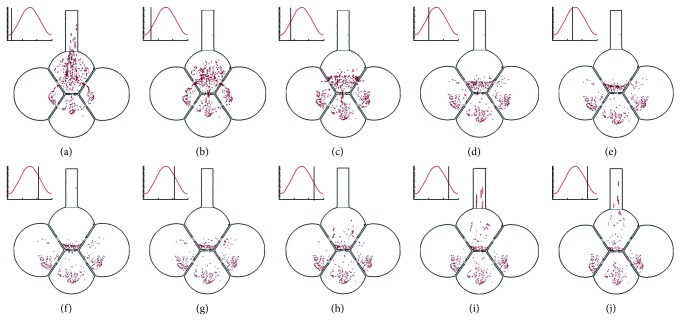
Instantaneous snapshots of particle positions in the 4-alveoli model during the second cycle after particle inhalation. All the remaining particles will deposit eventually due to particle interception or gravitational sedimentation. (a) *T* = 3.25 s. (b) *T* = 3.50 s. (c) *T* = 3.75 s. (d) *T* = 4.00 s. (e) *T* = 4.25 s. (f) *T* = 4.75 s. (g) *T* = 5.00 s. (h) *T* = 5.25 s. (i) *T* = 5.50 s. (j) *T* = 5.75 s.

Particle dynamics in the oscillating alveoli are visualized in Figures 3 and 4.  Figure 3 shows the snapshots of particle positions during the first cycle at ten different instants. Particles were inhaled into the geometry approximately at the beginning of inhalation. Depending on local velocities, the swarm of particles exhibits a parabolic pattern in the alveolar duct (*T* = 0.25 s) and a spherical shape after entering the top alveolus (i.e., Sac 1, *T* = 0.50 s). Around the middle of inhalation cycle (*T* = 0.75 and 1.0 s), particles start entering the adjacent alveoli through the pores of Kohn and deposit on the septal walls in Sac 1. In contrast, the particle fronts in the three adjacent alveoli only reached half of the airspace at the end of the first inhalation cycle (*T* = 1.25 s).

During expiration, particles gradually reversed their direction and started to move upward (*T* = 1.75–2.75 s). Particles that returned to Sac 1 from the three peripheral alveoli (Sacs 2–4) gained momentum due to the pores' accelerating effect. They mixed with the relativity slow-moving particles in the top alveoli, which enhanced particle dispersion. At the end of the first cycle, some particles were exhaled out of the alveoli, as displayed at *T* = 2.50–2.75 s. In principle, these particles would not be able to re-enter the geometry.

The instantaneous snapshots of particles during the second respiration cycle are shown in Figure 4 at ten instants. Similar as in the first cycle, the particle swarm oscillated with the expanding-contracting wall motion. However, a negligible number of particles was observed to leave the geometry. Instead, all these particles eventually deposited on the septal walls due to oscillatory convection and gravitational sedimentation. In the following sections, we would study the distribution of particles among individual alveoli as well as the influences of breathing conditions and pore size on these distributions.

**Figure 5:**
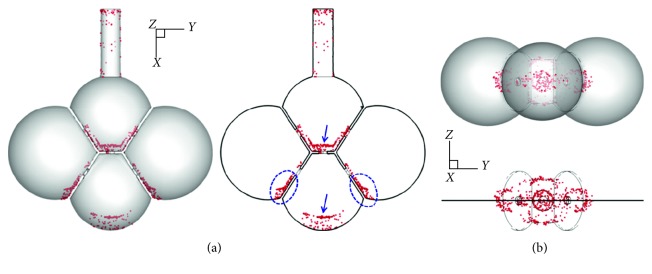
Surface deposition of 1 *µ*m particles in the 4-alveoli model with a pore size of 40 *µ*m: (a) side view and (b) bottom view.

Figure 5 shows the surface deposition pattern of 1 *µ*m particles. Most particles deposited on the septal walls of the top and two lateral alveoli, indicating that gravitational sedimentation is still the predominant deposition mechanism. It was noted that the deposition pattern in Figure 5 displayed the positions of the particles when they deposited on the moving walls of the alveoli; therefore, particle deposition was not limited to one surface but was possible on any surface in a range spanned by the oscillating wall motion.

From Figure 5, a large portion of particles deposited at the end of exhalation (wall contraction) when alveoli have the smallest volume. This was because that, near the end of the exhalation, gravitational sedimentation overtook the upward-moving momentum from the contracting wall. As a result, particles moved downward and collided with the upward-moving wall. Moreover, at the start of the subsequent inhalation, particles still outran the expanding walls and deposited there. As a result, a seemingly suspending layer of deposited particles was observed in the top and lower alveoli (solid arrow in Figure 5(a)). Considering the two lateral alveoli, no particles were observed on the distal walls, indicating a limited contribution from convective deposition. By contrast, most particles deposited on the interalveolar septal walls that bordered with the lower alveolus (i.e., Sac 4), with even more concentrated deposition in the lower position of the septal walls (dashed ellipse in Figure 5(b)).

**Figure 6:**
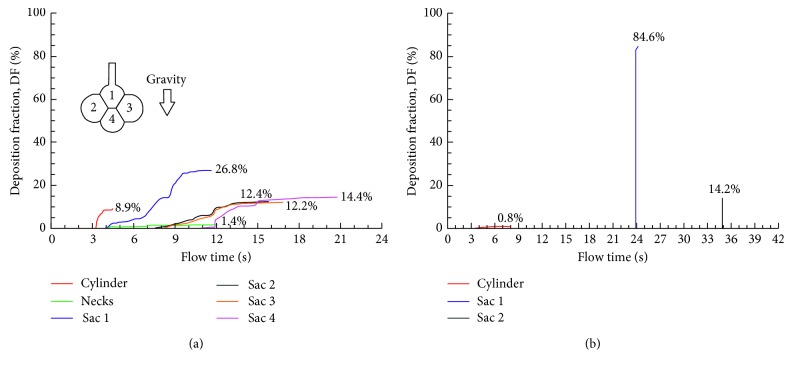
Cumulative deposition rate with time for 1 *µ*m particles in the 4-alveoli model: (a) dynamic model with rhythmic wall motions and (b) static model. Each respiration cycle has a period of 3.0 s.

Three major differences were observed when comparing temporal deposition profiles between dynamic and static models (Figure 6). First, the cumulative deposition rates were different, with 100% in the static model versus 76.2% in the dynamic model for 1 *µ*m particles. Second, the spatial distributions of particle deposition among alveoli were different. Heterogeneous doses were predicted in the dynamic model, with 26.8% in the upper alveolus (Sac 1), 14.4% in the lower alveolus (Sac 4), and about 12.3 ± 1% in each of the two lateral alveoli (Sac 2 and 3). In addition, there was also an appreciable fraction of particles that deposited in the alveolar duct (8.9%) and the canals of the pores (1.4%), as shown in Figure 6(a). By contrast, no particle deposited in the two lateral alveoli of the static model (Figure 6(b)). The third difference was the time required for deposition. It took about 24 seconds to start deposition in the static model, which was dictated by the alveolar size over particle settling velocity; while in the dynamic model, deposition started almost immediately after particles enter the alveoli. Furthermore, once the deposition started, it took only a short period time for the deposition to complete in the static model; in contrast, the deposition in the dynamic model took about three cycles to complete in the upper alveolus and more than four cycles in the lower and lateral alveoli. Further, a multistage staggering profile of the deposition fraction (DF) versus time was observed in each alveolus of the dynamic model. These three differences clearly demonstrated that analyses that neglected the dynamic nature of the alveoli would miss the particle behaviors in the acinar region.

**Figure 7:**
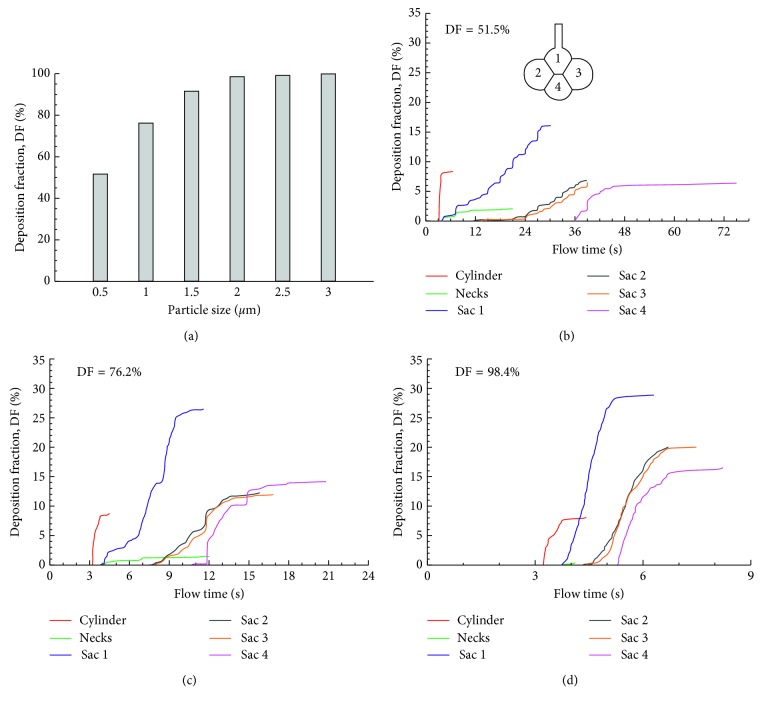
Comparison of particle deposition between different particle sizes in the 4-alveoli model: (a) total deposition fractions for 0.5–3 *µ*m particles and the time resolution of sectional cumulative deposition for (b) 0.5 *µ*m, (c) 1 *µ*m, and (d) 2 *µ*m. Deposition was quantified in each section of the model, that is, cylinder, the four alveoli (sacs), and the interalveolar pores (necks).

Deposition fractions for different sized particles are shown in Figure 7, which increases quickly with particle sizes. It is about 51.5% for 0.5 *µ*m particles and 76.1% for 1 *µ*m particles. For particles larger than 2 *µ*m, nearly all deposits are in the alveolar airspace (i.e., 98.4% for 2 *µ*m particles and 100% for 3 *µ*m particles). It is noted that, in this study, a particle bolus was inhaled into the geometry at the start of the inhalation. Particle boluses inhaled at later instants will have lower deposition rates [16].

To gain a better understanding of particle behaviors at different sizes, temporal variation of DFs was quantified in each section of the alveolar sacs (i.e., alveolar duct, four alveoli, and interalveolar pores, Figures 7(b)–7(d)). Similar to the dynamic case in Figure 6, heterogeneous and continuous particle deposition was observed for all sized particles, with the upper alveolus receiving the highest deposition. The time required for deposition was very different between different particle sizes. It took around 45 s (i.e., 48–3 in Figure 7(b)) for 0.5 *µ*m particles to complete the deposition, while it took 12 s (i.e., 15–3 in Figure 7(c)) for 1 *μ*m particles, 4 s for 2 *μ*m particles, and 2 s for 3 *μ*m particles. These decreases are due to the increasing particle settling velocity, which is proportional to the square of the particle diameter (i.e., *v*_settling_ = 18(*ρ*_p_ − *ρ*_f_)*gd*_p_^2^/18*μ*). Furthermore, deposition of 0.5 *µ*m particles started 21 s after administration in the two lateral alveoli and 33 s in the lower alveoli. By contrast, much shorter periods of time were needed for larger particles. For instance, it took 1 s for 2 *μ*m particles to start deposition in the lateral alveoli and 1.5 s in the lower alveoli (Figure 7(d)).

**Figure 8:**
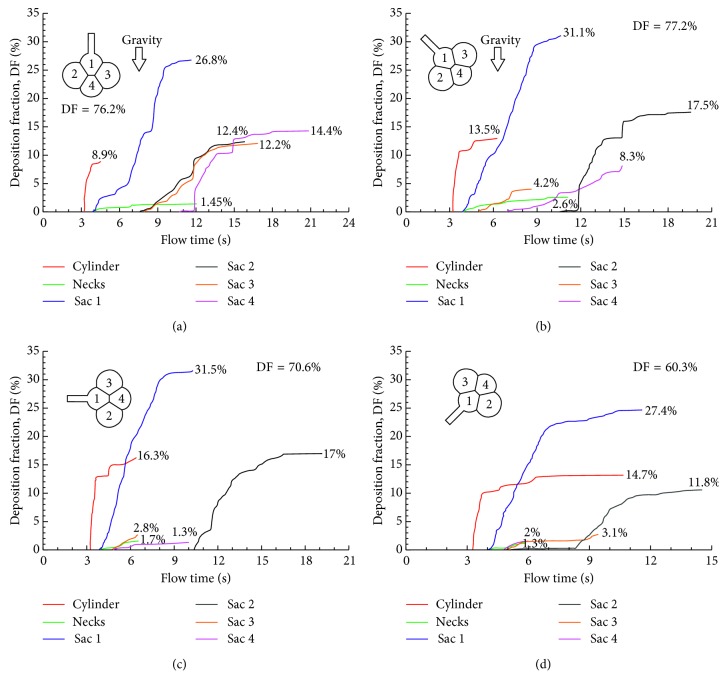
Comparison of the cumulative deposition with time for different gravity orientation angles in the 4-alveoli model: (a) 0°, (b) 45°, (c) 90°, and (d) 135° counterclockwise from the gravity.

The effects of gravity orientation angle on particle deposition in the alveolar sacs are shown in Figure 8. For the four cases considered (0°, 45°, 90°, and 135°), the total DF ranged from 60.3 to 77.2%. The highest subregional DF still occurred in Sac 1 (26.8–31.5%). However, the distribution of DFs in the other three alveoli changed significantly, depending on the alveolar orientation relative to the gravity. For instance, the DF in Sac 2 changed from 12.4% at 0° to ∼17% at 45° and 90° and 11.8% at 135°, while the DF in Sac 3 changed from 12.2% at 0° to 2∼4% at 45°–135°. Considering that the major deposition mechanisms herein were gravitational sedimentation and oscillatory convection, the orientation-induced DF variation was closely associated to the projected area normal to the gravity, as well as the convective penetration depth of particles into the distal alveoli. Similarly, DF in the alveolar duct was the highest at 90° (16.3%, Figure 8(c)) due to its largest projected area normal to the gravity and was lowest at 0° (8.9%, Figure 8(a)).

**Figure 9:**
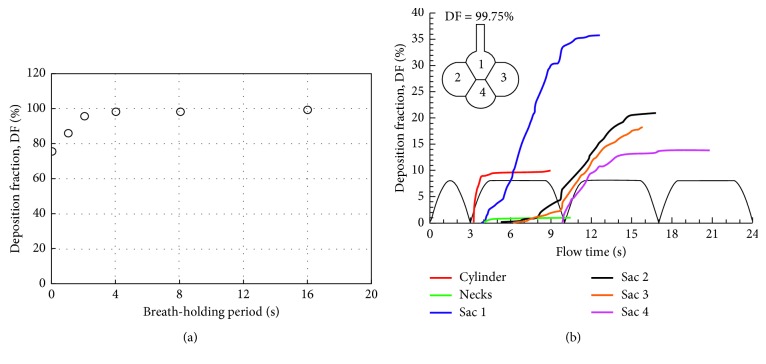
Comparison of total deposition rates for different breath-holding periods in the 4-alveoli model: (a) total deposition and (b) temporal deposition for 4 s breath-holding.

Breath-holding after inhalation can significantly enhance alveolar deposition.  Figure 9(a) shows the predicted DF of 1 *µ*m particles with different periods of breath-holding. Four seconds or longer hold of breath allowed all inhaled particles to deposit.  Figure 9(b) shows the temporal and spatial variation of DFs in the alveolar sacs for breath-holding of 4 seconds. Compared to the case without breath-holding (Figure 8(a)), subregional DF in Sac 1 increased significantly, for example, from 26.8% to 36.5%. Subregional DFs in the three peripheral alveoli also increased with the breathing-holding due to the prolonged action period of gravitational sedimentation relative to the particle dispersion from oscillatory convection.

**Figure 10:**
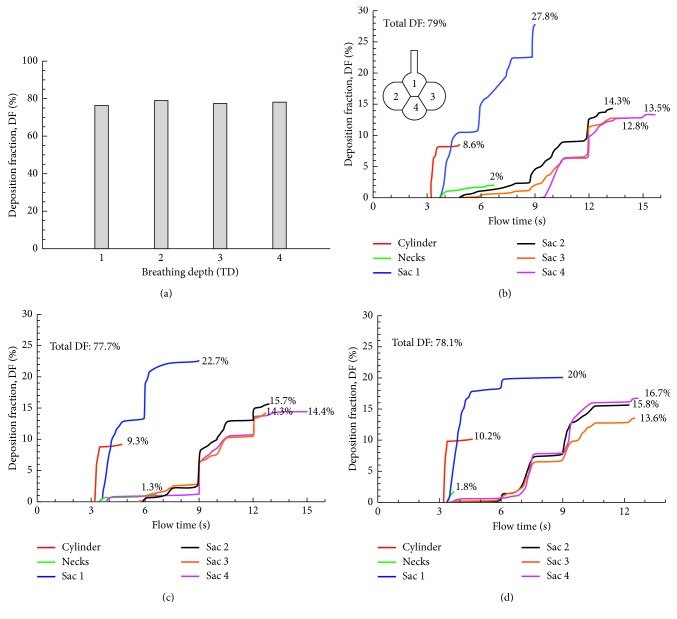
Comparison of particle deposition rates between different inhalation depths in the 4-alveoli model: (a) total deposition at different tidal volumes (*V*_T_) and the time resolution of sectional cumulative deposition for (b) 2 standard *V*_T_ (i.e., volume expansion factor: 0.466), (c) 3 standard *V*_T_ (i.e., volume expansion factor: 0.699), and (d) 4 *V*_T_ (i.e., volume expansion factor: 0.932). Each respiration cycle has a period of 3.0 s.

The effects of inhalation depth on alveolar deposition and its distribution in the 4-alveoli model are shown in Figure 10. Increasing the inhalation depth had a negligible effect on total DF. However, it notably changed particle distributions among alveoli, with deeper inhalations being associated with less heterogeneous subregional depositions. For instance, at higher tidal volumes, the DF in Sac 1 decreased while DF in Sac 4 increased, reducing the difference between them (Figures 10(b) vs.  10(d)). A subtle variation in DF was also noted in the alveolar duct, which increased slightly but persistently with increasing tidal volumes.

**Figure 11:**
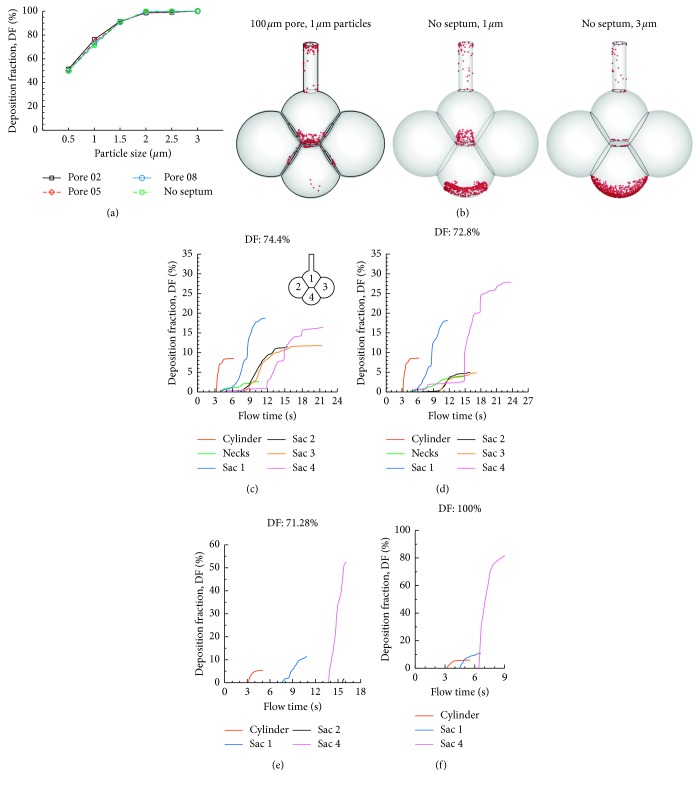
Pore size effects on alveolar deposition in the 4-alveoli model: (a) total deposition rates in the alveoli with different pore sizes, (b) surface deposition in different alveoli models, and the time resolution of sectional cumulative deposition for (c) pore size: 100 *µ*m, particles: 1 *µ*m, (d) pore size: 160 *µ*m, particles: 1 *µ*m, (e) no septum, particles: 1 *µ*m, and (f) no septum, particles: 3 *µ*m.

One significant issue of pulmonary pathology is the breakdown of interalveolar septal walls and associated collateral ventilation. To investigate their effects on acinar deposition, models with different sizes of septal apertures (pores) were simulated and compared in Figure 11(a). Surprisingly, total DF was found to be insensitive to the pore size. For all pore sizes considered (40 *µ*m, 100 *µ*m, 160 *µ*m, and no septal wall), the total DF for 1 *µ*m particles varied within a narrow range of 71.3∼74.4% (Figures 11(b)–11(e)). Dramatic changes in the spatial deposition distribution with the pore size were predicted. Considering the cases of pore size from 40 *µ*m (Figure 8(a)) to 160 *µ*m (Figures 11(c) and 11(d)), the DF in Sac 4 constantly increased with pore size, while DFs in the two lateral alveoli (Sac 2 and 3) decreased with pore size. This trend was reasonable because a larger pore allowed more particles to enter the lower alveolus (due to gravity) but at the same time, decreased the Venturi effect of pore aperture and therefore reduced the particle penetration depth to the lateral alveoli. When there was no septal wall, the alveolar sacs behave more like a single alveolus, as in Talaat and Xi [15], where particle deposition concentrated at the bottom of the alveolus (Figures 11(b) and 11(e)). This phenomenon was even more pronounced for 3 *µ*m particles, where the majority of particles deposited in the lower alveolus (Sac 4, Figures 11(b) and 11(f)).

**Figure 12:**
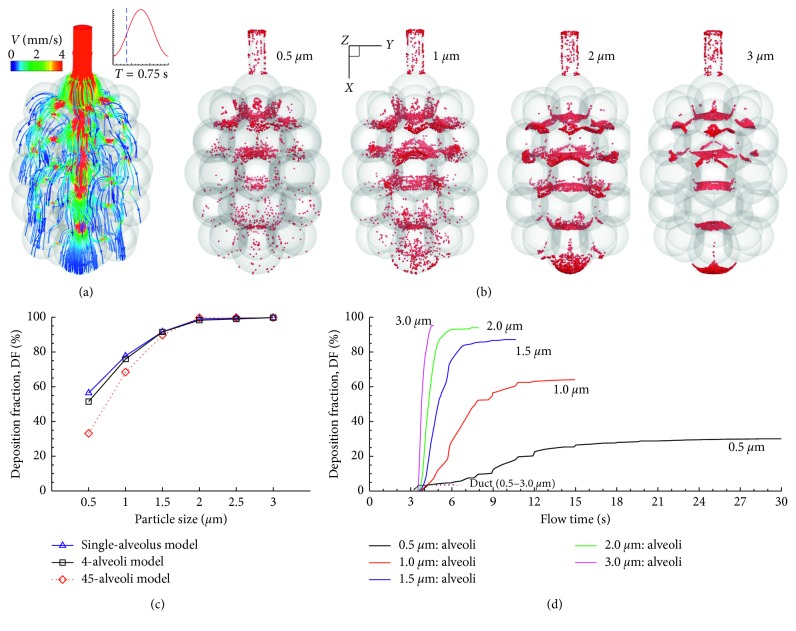
Airflow and particle deposition in the 45-alveoli model: (a) airflow, (b) surface deposition, (c) comparison of deposition rate between the 45-alveoli model and 4-alveoli and single-alveolus models, and (d) deposition with time.

Airflow and particle deposition in the 45-alveoli model was also simulated.  Figure 12(a) shows the inspiratory airflow in the 45-alveoli model. Complex flow fields are observed as the inhaled airflow enters the alveolar sacs sequentially from top to bottom and from central to peripheral. Airflow is stronger in the axial direction (i.e., positive *x*-direction, Figure 12(a)) and decreases progressively in the lateral compartments. Surface deposition of inhaled particles is shown in Figure 12(b). Overall symmetric surface deposition patterns are observed. Due to the multiple alveolar generations retained in this model, particle deposition exhibits a cascading pattern for all particle sizes (0.5–3.0 *µ*m) considered (Figure 12(b)). However, deposition is more dispersed for small particles and more concentrated for large particles.

Figure 12(c) compares the deposition fractions as a function of particle size between the 45-alveoli and 4-alveoli models, as well as the single-alveolus model in [15]. Surprisingly, for 0.5–1.5 *µ*m particles, lower deposition fractions were predicted in the complex 45-alveoli model than the highly simplified 4-alveoli and single-alveolus models, indicating a negative correlation of acinar deposition with the number of alveoli retained in the model. The cumulative deposition profiles with time were displayed in Figure 12(d) for 0.5–3.0 *µ*m particles. The time required for complete deposition decreased dramatically from 0.5 *µ*m to 3.0 *µ*m (Figure 12(d)).

**Figure 13:**
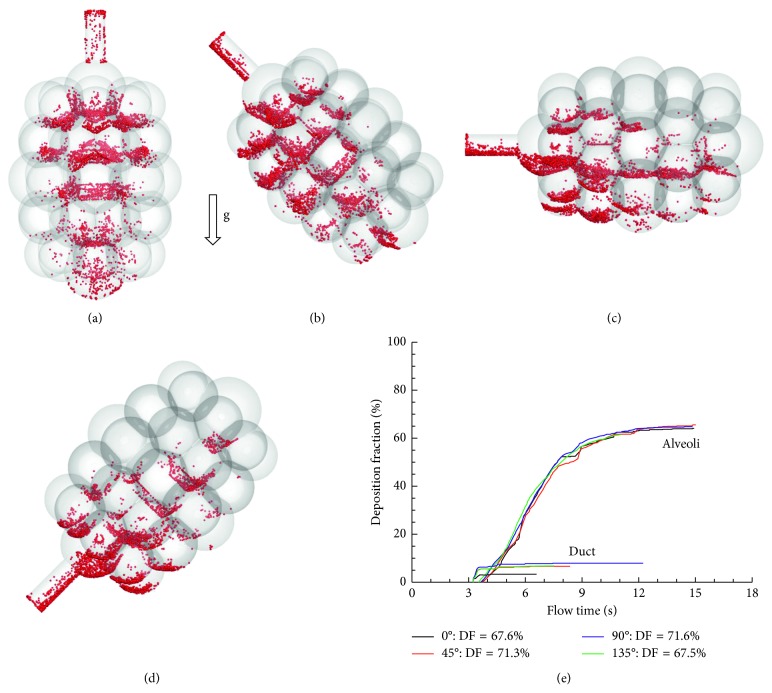
Comparison of the cumulative deposition with time for different gravity orientation angles in the 45-alveoli model: (a) 0°, (b) 45°, (c) 90°, and (d) 135° counterclockwise from the gravity, and (e) deposition.

Effects of gravity orientation angle on particle deposition in the 45-alveoli model are shown in Figure 13. It appears that the total DF is not sensitive to the gravity orientation angle, as demonstrated by the close similarity of temporal deposition profiles among the four angles considered (i.e., 0°, 45°, 90°, and 135° counterclockwise from the gravity, Figure 13). The spatial deposition distributions, however, exhibit high levels of heterogeneity. In contrast to the symmetric distribution in the 0° case (Figure 13), particles in the other three models deposit preferentially in the central alveoli or alveoli along the gravity (Figures 13–13). Particle deposition decreases quickly in the distal alveoli; very few particles are observed in the alveoli opposite to the gravity direction (Figures 13–13).

**Figure 14:**
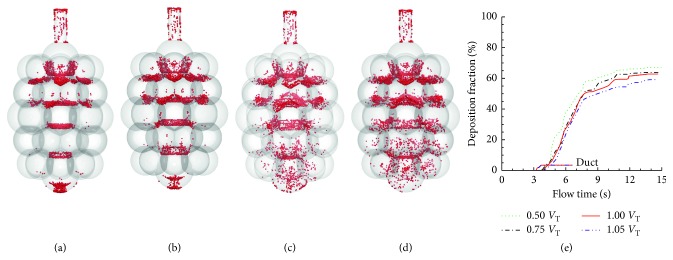
Comparison of particle deposition with different breath depths in the 45-alveoli model: (a) total deposition vs. inhalation depth and the time resolution of sectional cumulative deposition for (b) 0.5 standard *V*_T_ (i.e., volume expansion factor: 0.117), (c) 0.75 standard *V*_T_ (i.e., volume expansion factor: 0.175), (d) 1.05 standard *V*_T_ (i.e., volume expansion factor: 0.245), and (e) 1.1 standard *V*_T_ (i.e., volume expansion factor: 0.256).

Effects of inhalation depth on particle deposition in the 45-alveoli model are shown in Figure 14. The inhalation depth ranges from 0.5 to 1.05 standard *V*_T_ (i.e., one standard *V*_T_ = 0.233 FRC), and the particle size is 1 *µ*m. Very different deposition patterns are noted for different inhalation depths. At very shallow breath (i.e., 0.5 standard *V*_T_ or volume expansion ratio = 0.117), particles concentrate in the central alveoli along the gravity direction while very few particles deposit in the peripheral alveoli. As the breath depth increases, particle deposition becomes progressively more dispersed (Figures 14–14). This is caused by the deeper ventilation and stronger flow irregularity at a higher flow rate, both of which will lead to enhanced particle mixing. This phenomenon is most pronounced in the highest breath depth considered (1.05 *V*_T_), where particles are spread in all compartments of the 45-alveoli model geometry (Figure 14). Figure 14 shows the cumulative deposition with time for 1 *µ*m particles at varying inhalation depths. Similar temporal profiles are observed among the four inhalation depths considered, all with a steep slope during the first two cycles and approaching asymptotic thereafter. The final DF slightly increases with the breath depth, for instance, from 62.6% at 0.5 *V*_T_ to 70.5% at 1.05 *V*_T_ (Figure 14).

## 4. Discussion and Summary

A systematic study of acinar deposition of inertial particles was conducted in a well-defined 4-alveoli model and a more realistic 45-alveoli model. Airflow and particle dynamics driven by oscillating wall motions were visualized. A parametric study of various respiratory and structural factors was conducted, which included alveolar wall kinematics, particle size, model orientation, breath-holding, inhalation depth, and size of interalveolar pores.

Similar to the single-alveolus model as considered in [15], oscillatory wall motion was essential for the multialveoli models to capture particle dynamics and acinar deposition. A static multialveoli model that neglected oscillatory wall motions failed to predict neither the deposition fraction nor the particle lifetime before deposition. Much longer time was needed for particles to start deposition in a static alveolar model (about seven respiration cycles, Figure 5) than in a dynamic model (less than three cycles) [15]. This observation called into question of the using aerosols to estimate the alveolar size *in vivo* [48].

In comparison to a single-alveolus model [15], interesting differences were observed in multiple-alveoli (i.e., 4-alveoli and 45-alveoli) models. One difference between the single-alveolus model and multialveoli model is the time to start deposition after inhalation. In a terminal single-alveolus model, inhaled particles cannot reach the airway wall during the first inhalation cycle due to the resident air, as observed both experimentally by Berg et al. [49] and in numerical studies by Sera et al. [50] and Talaat and Xi [15]. It took about three breathing cycles for 1 *µ*m particles to start deposition and even longer for submicron particles [15]. In contrast, particle deposition in multialveoli models started during the first inhalation cycle. As shown in Figures 3(d) and 3(e), inhaled particles reached the septal walls of the upper alveolus (Sac 1) around the middle of the cycle. Due to the interalveolar communication in multialveoli models, all residual air in Sac 1 was displaced into the peripheral alveoli, transporting particles to Sac 1's septa; while the residual air in the single-alveolus model remained in the airspace during wall expansion, which restrained particles' forward motion and kept particles from reaching the alveolar wall.

The acinar deposition was found to be considerably sensitive to the number of alveoli retained in the model. In this study, the total DF for 0.5–1.5 *µ*m particles was lower in the 45-alveoli model than that in the 4-alveoli model, which in turn was slightly lower than that in the single-alveolus model. This might be counterintuitive at first sight, as complex structures are generally expected to capture more inhaled particles. This is mostly true for throughout flows with inlets and outlets. However, the terminal acinar region is a blind-end airspace, where airflow is driven by the rhythmical wall motion, which enters and exits the geometry through the same inlet. Some particles will be exhaled out of the geometry during expiration, leading to incomplete deposition. The escaping particles are primarily due to dispersion. Particles that remain in the geometry will eventually deposit either by convection impaction or gravitational sedimentation. Concerning the geometrical complexity effect, the 45-sac model will filter more particles for a given period of time, but meanwhile causes much stronger dispersion, overall giving rise to a lower total DF.

Second, self-similarities exist of particle dynamics in different generations of alveolar sacs. Particles in the central alveoli are more likely to deposit via convective impaction or interception, whereas particles in the peripheral alveoli will deposit via sedimentation, where the flow is slower. For a given tidal volume (i.e., 23.3% FRC), the 45-alveoli model draws in about ten times of air than the 4-alveoli model, inducing stronger inertia impaction in the central alveoli and higher particle deposition. As the inhaled flow bifurcates into the more distal compartments, the inertial impaction effect quickly decreases yielding much lower deposition. Overall, the total DF in the 45-alveoli model can be lower than a simpler 4-alveoli or single-alveolus model.

Particle deposition of these models (i.e., 1-, 4-, and 45-alveoli) reacted differently to the model orientation relative to the gravity. The DF was found to be relatively sensitive to the gravity orientation angle in the single-alveolus model (62.6%–80.0% from 0° to 135°) [15] and the 4-alveoli model (60.3%–77.2% from 0° to 135°), but was insensitive in the 45-alveoli model (67.5%%–71.6% from 0° to 135°). The lowest DF occurred at 135° for all of the three models considered. Considering that gravitational sedimentation was one dominating deposition mechanism, the above variation was most likely attributed to area ratio of the duct inlet over the projected area of the alveoli normal to the gravity, which decreased as the model become more complex. Accordingly, it was anticipated that particle dose in more complex acinus should be orientationally insensitive too. A similar observation was also reported in Khajeh-Hosseini-Dalasm and Longest [22] that acinar deposition was not affected by gravity orientation angle when the number of alveolar duct generations was more than three.

The inhalation depth was found to have an insignificant effect on the acinar DF in both the single-alveolus model [15] and the two multialveoli models herein. However, inhalation depth significantly altered the spatial distribution of the particle deposition, with more deposition rates in peripheral alveoli at deeper inhalations. A higher inhalation depth means a higher airflow speed and stronger wall-flow-particle interaction, which further lead to an enhanced deposition from convection and interception, as well as an intensified particle dispersion. As discussed earlier, particle dispersion can cause more particles to escape the geometry during exhalation. Overall, the acinar DFs remain similar between different inhalation depths for a given acinar model. It is acknowledged that the acinar deposition fraction presented in this study was calculated as the number of particles deposited in the alveoli over the number of particles entering the alveolar model. The fraction of orally inhaled particles that entered the alveolar airways was not considered. Increasing inhalation depth will convey more particles to the alveolar airways and lead to higher doses in the acinar region.

In a recent study, Hofemeier et al. [23] also reported that that variance in acinar heterogeneity had little effect on total deposition. When comparing the DF between different acinar models (Figure 12(c)), the DF-particle profiles exhibit a similar asymptotic pattern among the three models, despite the difference in DF magnitude for particles ranging from 0.5 to 1.5 *µ*m. This similarity was to a large extent due to the similar oscillating flows and the associated particle dynamics. Taken other similarities altogether, this agreement suggests a possibility of a generic deposition correlation to estimate the dosimetry in the intricate alveoli using relatively simple geometries even though what level of geometry complexity would be sufficient is yet to be determined. It is emphasized that Khajeh-Hosseini-Dalasm and Longest [22] pursued this question in multigeneration space-filling models with no septal walls and reported a relatively constant acinar deposition rate when more than three alveolar duct generations were retained in the acinar model. Furthermore, correlations of alveolar dose were proposed for different ventilation waveforms such as quick-and-deep and slow-and-deep inhalations [22].

Results of the airflow and particle deposition in alveoli with different pore sizes have meaningful implications in emphysema, which is featured by pore size increase and septum destruction. Varying the pore size was found to exert an insignificant impact on the alveolar deposition, indicating that the emphysematous terminal sacs might receive a similar amount of inhaled aerosols. It is noted that the above results were obtained with the remaining parameters being kept constant, while an emphysematous alveolus might also experience other changes, such as size increase, more compliant (i.e., longer exhalation time), and structure remodeling.

Besides interalveolar pores, collateral ventilation can also occur via bronchiole-alveolar communications (Lambert's channels), interbronchiolar communications (Martin's channels), and even interlobular respiratory bronchioles, depending on the alveolar location and emphysema severity [3]. This study focused on the terminal alveolar sac, where the bronchiole-alveolar and interbronchiolar channels are scarce and the interalveolar pores are prevalent [3, 51]. As a result, results of this study should only be applicable in the terminal alveoli or apical acinar regions where pores of Kohn were found in greatest numbers [3].

Other limitations of this study include simplified model geometry, ideal breathing conditions, noncontinuous aerosol inhalation, and one-way (wall-flow-particle) interaction. For simplicity, particle charge [52, 53], size [54], and hygroscopy effect [55] were also excluded. *In vivo* pulmonary alveoli have complicated morphology as revealed by histology and microscopy studies [49, 56–58], which appear as a polyhedral complex with varying-sized alveoli grouped in a fractal form [59]. In this study, both the 4-alveoli and 45-alveoli models were constructed from regular geometries such as a cylindrical duct, spheres, and circular pores. Anatomically, the interalveolar septal wall was demonstrated to have a variable thickness from the alveolar mouth to distal walls [60]; a constant wall thickness was assumed herein for computational simplicity. Likewise, there was no more than one pore in one septum in this study, while there can be one to seven pores in life conditions [61]. Even though scanning electron microscope (SEM) evidence has confirmed that pores of Kohn are normal structures in healthy lungs, it is not clear whether they are open all the time or are mostly covered by surfactant that ruptures during expansion or at high differential pressures. Because SEM samples are *ex vivo*, to the authors' knowledge, there is no literature that tracked the size and shape of *in vivo* pores in human lungs, despite recent attempts that utilized confocal microscopy [4] and optical coherence tomography (OCT) imaging to visualize alveolar structure dynamics [62] in mouse models. Moreover, surface tension of the fluids/surfactant mixture lining the alveolar wall varies during the expansion and contraction of the alveoli (roughly proportional to the surface area) [63]. Along with the nonlinear alveolar tissue elasticity, this surface tension variation further complicates the alveolar kinematics, for instance, by slowing down or constraining the wall stretching as it approaches the end of inhalation [64]. However, essential geometrical features of the alveoli were retained in these two models, such as the septa between alveoli and the pores in the septa, both of which had been neglected in previous numerical studies. The sphere was also naturally reshaped into a polyhedron when several spheres cross-cut each other, as displayed by the polyhedrons inside the 45-alveoli model, as well as the semipolyhedrons in the outer layers of both models (Figure 1). More importantly, well-defined shape and size allowed controlled parametric studies and hence identification of major factors that dictate airflow and particle deposition in alveolar sacs. The second physiological parameter to be improved is the breathing profile, which can have different waveforms and inhalation : exhalation (I : E) ratios. In life conditions, a normal breathing in a healthy subject generally has an I :  E ratio of 1 : 2. [65] In emphysematous patients, the loss of alveolar elasticity leads to even longer exhalation time [8]. Compared to an I : E ratio of 1 : 1, this means a longer period of exhalation, a slower expiratory speed, a further downward motion, and therefore a higher deposition rate. This scenario should be close to the case with a postinhalation breath-holding of 1 or 2 seconds. Thirdly, particles were inhaled only at the beginning of the inhalation, and results of this study cannot be applied in scenarios with continuous aerosol exposures. Lastly, kinematics of the alveolar wall was modeled based on experimentally measured chest motions [37], and the amplitude of the alveolar wall motion was based on the tidal volume [66]. Direct measurements of *in vivo* alveolar motion using the imaging method should be used in future studies. It is also noted that pores of Kohn is 2–15 *µ*m in diameter in normal healthy lungs [2, 61], but can constantly increase in size till septal wall breakdown in emphysematous alveoli [59]. Further studies of the influences of smaller and multiple pores on acinar flow and particle deposition are needed.

In Summary, temporal and spatial deposition variations in multialveoli pore-communicated acinar models were numerically investigated under the influences of various physiological factors. Specific findings are listed as follows:

1. Collateral ventilation existed in multialveoli acinar models
2. Heterogeneous deposition distributions were found among alveoli, with the highest deposition in the central alveoli and decreasing deposition in more peripheral alveoli
3. The acinar deposition was highly sensitive to particle size; for particles that were larger than 2 *µ*m and administered at the beginning of the inhalation, nearly 100% alveolar deposition fraction (i.e., particles deposited in the alveolar model over particles entering the alveolar model) was predicted
4. The number of alveoli retained in the model affected the total deposition, with the 45-alveoli model having lower deposition than the 4-alveoli and single-alveolus models
5. The size of the pores of Kohn, inhalation depth, and gravity orientation angle had insignificant effects on the acinar deposition fraction but had a dramatic impact on the spatial distribution of particle deposition among alveoli

## Data Availability

The data used to support the findings of this study are available from the corresponding author upon request.
